# Melanin distribution from the dermal–epidermal junction to the *stratum corneum*: non-invasive in vivo assessment by fluorescence and Raman microspectroscopy

**DOI:** 10.1038/s41598-020-71220-6

**Published:** 2020-09-01

**Authors:** B. P. Yakimov, E. A. Shirshin, J. Schleusener, A. S. Allenova, V. V. Fadeev, M. E. Darvin

**Affiliations:** 1 Faculty of Physics, M.V. Lomonosov Moscow State University, 1-2 Leninskie Gory, Moscow, Russia 119991; 2 Medical Research and Education Center, M.V. Lomonosov Moscow State University, Lomonosovsky Prospect 27/10, Moscow, Russia 119991; 3 Institute for Regenerative Medicine, Sechenov First Moscow State Medical University, Trubetskaya 8-2, Moscow, Russia 119991; 4 Division of Immune-Mediated Skin Diseases, Sechenov First Moscow State Medical University, Trubetskaya 8-2, Moscow, Russia 119991; 5 Institute of Spectroscopy of the Russian Academy of Sciences, Fizicheskaya Str., 5, 108840 Troitsk, Moscow, Russia; 6 Department of Dermatology, Venerology and Allergology, Center of Experimental and Applied Cutaneous Physiology, Charité – Universitätsmedizin Berlin, Corporate Member of Freie Universität Berlin, Humboldt-Universität zu Berlin, and Berlin Institute of Health, Charitéplatz 1, 10117 Berlin, Germany

**Keywords:** Biophotonics, Fluorescence spectroscopy, Raman spectroscopy, Confocal microscopy, Biological fluorescence

## Abstract

The fate of melanin in the epidermis is of great interest due to its involvement in numerous physiological and pathological processes in the skin. Melanin localization can be assessed ex vivo and in vivo using its distinctive optical properties. Melanin exhibits a characteristic Raman spectrum band shape and discernible near-infrared excited (NIR) fluorescence. However, a detailed analysis of the capabilities of depth-resolved confocal Raman and fluorescence microspectroscopy in the evaluation of melanin distribution in the human skin is lacking. Here we demonstrate how the fraction of melanin at different depths in the human skin in vivo can be estimated from its Raman spectra (bands at 1,380 and 1,570 cm^−1^) using several procedures including a simple ratiometric approach, spectral decomposition and non-negative matrix factorization. The depth profiles of matrix factorization components specific to melanin, collagen and natural moisturizing factor provide information about their localization in the skin. The depth profile of the collagen-related matrix factorization component allows for precise determination of the dermal–epidermal junction, i.e. the epidermal thickness. Spectral features of fluorescence background originating from melanin were found to correlate with relative intensities of the melanin Raman bands. We also hypothesized that NIR fluorescence in the skin is not originated solely from melanin, and the possible impact of oxidized species should be taken into account. The ratio of melanin-related Raman bands at 1,380 and 1,570 cm^−1^ could be related to melanin molecular organization. The proposed combined analysis of the Raman scattering signal and NIR fluorescence could be a useful tool for rapid non-invasive in vivo diagnostics of melanin-related processes in the human skin.

## Introduction

Melanin, the pigment mainly responsible for skin color, participates in numerous physiological processes. Melanin exhibits photoprotective, antioxidative, and, in some cases, photosensitizing properties^1^. It is also directly involved in the life cycle of the healthy and pathological epidermis, including such a severe malignancy as melanoma^2^. Being heterogeneous end-products of the complex transformation of L-tyrosine^3^, melanins can be classified into several chemically distinct subtypes, of which eumelanin (black and brown) and pheomelanin (yellow-reddish) are present in the human epidermis^4,5^. In general, the structure of natural melanin is a complicated issue, as natively occurring melanin is a mixture of different pigment subtypes produced from the same precursor as a result of “mixed melanogenesis”^6^.

Melanin is produced in melanocytes—the cells, which at normal conditions are localized in the basal layer of the epidermis. Packed into melanosomes, melanin is transferred to the nearby keratinocytes: on average, a single melanocyte and ≈ 30–40 surrounding keratinocytes compose an epidermal melanin unit, and this ratio is preserved in different skin phototypes^7^. The subtype of melanin (ratio of eu- and pheo-melanin) and amount of melanin, as well as the amount, packing degree and size of the melanosomes contribute to the formation of the skin color. However, a complete understanding of melanin transfer mechanisms, its further distribution in the epidermis and degradation pathways remains elusive. For instance, melanin distribution was considered to be regulated by melanosomes degradation via keratinocytes’ autophagic activity^8^. However, in later works, autophagy was demonstrated not to have a substantial effect on skin pigmentation^9^, and melanosomes were shown to be non-degradative^10^. Other mechanisms like inherent asymmetric localization of melanosomes between dividing keratinocytes could be potentially involved in a complex inhomogeneous distribution of melanin in the epidermis^11–13^. Hence, precise assessment of melanin distribution in different epidermal layers is required to study fundamental processes and identify melanin-related abnormalities in norm and pathology.

A standard tool for melanin visualization in the skin is the histochemical staining. This procedure, however, is prone to false-positive results: for instance, observation of “melanin dust” (degraded melanin fragments) in the *stratum corneum* with this approach was shown to be an artifact caused by non-specific reduction of silver by non-melanin compounds in the skin^13,14^. The use of other staining agents, e.g. based on immunohistochemistry, allows for specific labeling of melanogenesis- and melanosome-specific proteins, but these methods are also highly labile^14^. A common disadvantage of histological studies is its invasiveness, which imposes restrictions on the number of patients involved in the investigation, the required time of analysis, and the inability to examine the same area of skin multiple times. Hence, optical techniques have emerged as a new generation of methods for non-invasive diagnostics of the skin. In this regard, the unique optical properties of melanin made it a perfect target for ex vivo and in vivo imaging (Table 1).

**Table 1:** Summary on non-invasive optical methods used for quantification, imaging and structural characterization of melanin in the skin

Optical method	Endogenous contrast	Application	References
Single-photon excited NIR fluorescence	Single-photon excited NIR fluorescence of melanin	Bulk and depths resolved measurements of melanin	^15–20^
Diffuse reflectance spectroscopy	Broadband absorption of melanin in visible and NIR spectral range	Assessment of bulk concentration of cutaneous melanin with ≈ 1 mm resolution	^18^
Two-photon excited fluorescence lifetime imaging (FLIM)	Short fluorescence lifetime (< 0.2 ns) of melanin and quasi-selective excitation at ca. 800 nm	Imaging of melanin in the basal layer with submicron resolution	^25–27^
Confocal laser-scanning microscopy	Enhanced elastic scattering on melanosomes	Imaging of melanin in the basal layer with submicron resolution	^32–34^
Pump-probe microscopy	Time-resolved excited-state absorption and ground state bleaching of melanin	Imaging of melanin subtypes (i.e. eumelanin and pheomelanin), differentiation between aggregation modes of melanin oligomers, evaluation of malignancy	^24,28–31^
Optical coherence tomography	Enhanced elastic scattering on melanosomes	Assessment of melanin near the basal layer with ≈ 10 µm resolution	^35,36^
Optoacoustics	Broadband absorption of melanin in visible and NIR range	Bulk measurements and depth-resolved localization with ≈ 10 µm resolution	^37–39^
Raman spectroscopy	Features of Raman scattering spectrum of melanin, proteins and lipids in 1,000–1,800 cm^−1^ region	Bulk quantification of melanin and depth-resolved imaging (submicron resolution) of melanin in vivo. Assessment of biochemical properties of melanin in vitro. Evaluation of malignancy	^40–44^

Unlike the other endogenous chromophores in the skin, melanin exhibits a specific broadband absorption, which decreases exponentially from short (UV) towards long (NIR) wavelengths. The long-wavelength absorption is accompanied by a discernible red/NIR excited fluorescence, and melanin is considered to be the major source of NIR fluorescence in the skin. That is, quantification of melanin in the skin can be performed by single-photon NIR-excited fluorescence imaging^15–20^. Moreover, melanin fluoresces upon two-photon excitation, allowing for its in vivo assessment and 3D imaging^21,22^. However, the fluorescence intensity itself does not provide specific information on the melanin type and its environment^23,24^. Additional contrasting of melanin can be achieved using fluorescence lifetime imaging microscopy (FLIM), as melanin exhibits a distinctive fast (< 0.2 ns) fluorescence decay^25–27^. Next, using the pump-probe imaging technique, one may differentiate between eumelanin and pheomelanin at the dermal–epidermal junction to assess the metastatic potential of melanin lesions and characterize the packing of melanin oligomers inside the aggregates^28–31^. Other optical techniques, including reflectance confocal laser scanning microscopy^32–34^ and optical coherence tomography^35,36^ may help in the detection of pathological melanocytic lesions. The long-wavelength absorption tail also allows the use of the photoacoustic signal to quantify melanin in the skin^37–39^.

Melanin exhibits a characteristic Raman spectrum. Multiple bands may contribute to the formation of the Raman spectrum of melanin in the 1,000–1,800 cm^−1^ range: the band at 1,220 cm^−1^, which corresponds to the stretching vibrations of the phenolic C–OH and C–O stretching in carboxylic acids, the 1,340 cm^−1^ band of the C–N stretching of the indole, the band with a maximum at 1,390 cm^−1^ produced by the C=C “breathing” vibrations of the aromatic structures (A_1g_ symmetry, similar to the D-band in disordered graphite). Also, the two bands at 1562 and 1598 cm^−1^, attributed to the stretching mode of sp^2^ hybridized carbon of C=C and E_2g_ mode of C–C vibrations of the aromatic ring in the indole structure of eumelanin, contribute to the observed Raman spectrum^40^. Despite this large number of bands, they are poorly resolvable due to the heterogeneous nature of melanin, and their superposition results in just two broad bands with maxima at ≈ 1,380 and ≈ 1,570 cm^−1^ and FWHMs of ≈ 200 and 150 cm^−1^, respectively. Depending on the excitation wavelength, the structural arrangement of melanin and its biochemical environment, the amplitude and positions of these bands may vary, allowing the characterization of melanin in vitro and quantification of melanin in vivo^40–44^.

In this work, we focused on a combined application of confocal Raman and fluorescence microspectroscopy to determine the depth-resolved melanin content in the human skin in vivo. Currently, a detailed analysis of the relationship between NIR-excited fluorescence and the molecular properties of melanin, which are manifested in the Raman spectra, is lacking in the literature. Hence, we made use of confocal Raman microspectroscopy to disentangle the impacts of fluorescence and Raman signals from melanin in order to investigate their diagnostic capabilities and studied the interconnections between the features of the Raman and fluorescence spectra of melanin in the human skin in vivo.

## Materials and methods

### Volunteers

A total of 10 healthy volunteers (2 male, 8 female) aged from 22 to 60 years (average 37.5 years) with skin type II (5 participants) and III (5 participants) according to the Fitzpatrick classification were involved in the study. All volunteers did not use any skincare products on the inner forearms at least 72 h and did not take a bath or shower at least 4 h before the beginning of the experiments. Nine volunteers involved in the study reported the absence of sunbathing at least 3 months before the experiment; the volunteers with a high fraction of melanin were naturally tanned. One volunteer (skin type III) experienced sun exposure for three days before the measurements.

All volunteers had given written informed consent. A positive vote has been obtained from the ethics committee of the Charité – Universitätsmedizin Berlin and the experiments were in accordance with the principles of the declaration of Helsinki as revised in 2013.

### Confocal Raman microspectroscopy (CRM): measurement parameters

To determine the possibilities of melanin localization in different epidermal layers, 6 depth-resolved Raman spectra (from 0 to 60 µm depth with increments of 2 µm) were measured for each of the 10 volunteers in vivo on the volar forearm using the confocal Raman microscopy (CRM) instrument (Model 3510 SCA, RiverD, Rotterdam, The Netherlands). CRM allowed measuring Raman and fluorescence signals simultaneously from the skin with a spatial resolution of less than 5 µm. The spectra were recorded in the fingerprint (FP: 400–2,200 cm^−1^) and high wavenumber (HWN: 2,500–4,000 cm^−1^) regions upon excitation at 785 (20 mW) nm and 671 nm (17 mW), respectively. The acquisition times for the FP and HWN spectra were 5 and 1 s, resulting in 3 J/cm^2^ and 0.5 J/cm^2^ irradiation dose per a single measurement, respectively. The principal scheme of the utilized CRM could be found elsewhere^45^.

The skin surface position was determined as the position where the intensity of the Amide I band calculated as the average intensity in the 1,550–1,720 cm^−1^ range after fluorescence background subtraction similar to the procedure reaches half of its maximum as it was proposed in the work of Choe et al.^46^. All data analysis was performed using a custom-made program based on the Python programming language with Matplotlib, NumPy, Pandas, Scikit-learn, SciPy libraries.

### Fluorescence background subtraction

Since melanin is considered to be the major contributor to the red/NIR-excited fluorescence in the human epidermis, the depth distribution of NIR fluorescence intensity was evaluated. To quantify the intensity and spectral properties of fluorescence emission and to extract the Raman signal, fluorescence background was evaluated by fitting the spectrum with a 2nd order polynomial function in the FP range, where the 620–700 cm^−1^ and 1900–2,200 cm^−1^ ranges were used for the interpolation of the fluorescence spectrum due to the absence of intense Raman bands. An analogous approach was previously used to assess and subtract the fluorescence background in the FP range in spectra with high melanin content^41^. The choice of 2nd order polynomial function as an estimator for fluorescence background is determined by the fact that on the one hand, it provides reasonable quality of fit and, on the other, provides a simple estimation on spectral features of fluorescence. Interpolation of the fluorescence background in the HWN range was made using piecewise-weighted-least squares fitting method, where 2,600–2,810 cm^−1^ and 3,800–3,900 cm^−1^ ranges were used for fluorescence estimation, proposed in^47^.

### Spectra normalization

To adjust for the fact that intensity of both Raman and fluorescence signal is attenuated due to scattering and absorption of the optical signal by tissues with an increase of scanning depth, we additionally normalized the Raman spectra obtained after background subtraction by the averaged protein-related Raman intensities in 1,550–1,720 cm^−1^ and 2,800–3,000 cm^−1^ ranges, respectively. This normalization procedure is used to monitor concentrations of various skin compounds relative to the concentration of proteins both estimated by intensities of Raman bands and was used in investigations of the dermis^48^.

### Influence of fluorescence photobleaching

The fluorescence intensity is prone to photobleaching, so its signal might reduce over time. As the Raman bands are not subject to photobleaching effect in comparison to fluorescence^49^, the ratio between fluorescence and Raman band intensities might change over time. However, according to^49^ at skin depths of 20–50 µm average fluorescence decay lifetime upon bleaching is of the order of 30 s for excitation at 785 nm and 22.0 mW laser power. Thus we hypothesize, that 1 and 5 s exposure time did not affect fluorescence intensity significantly.

## Results

### Evaluation of NIR fluorescence of the human skin in vivo

**Figure 1:**
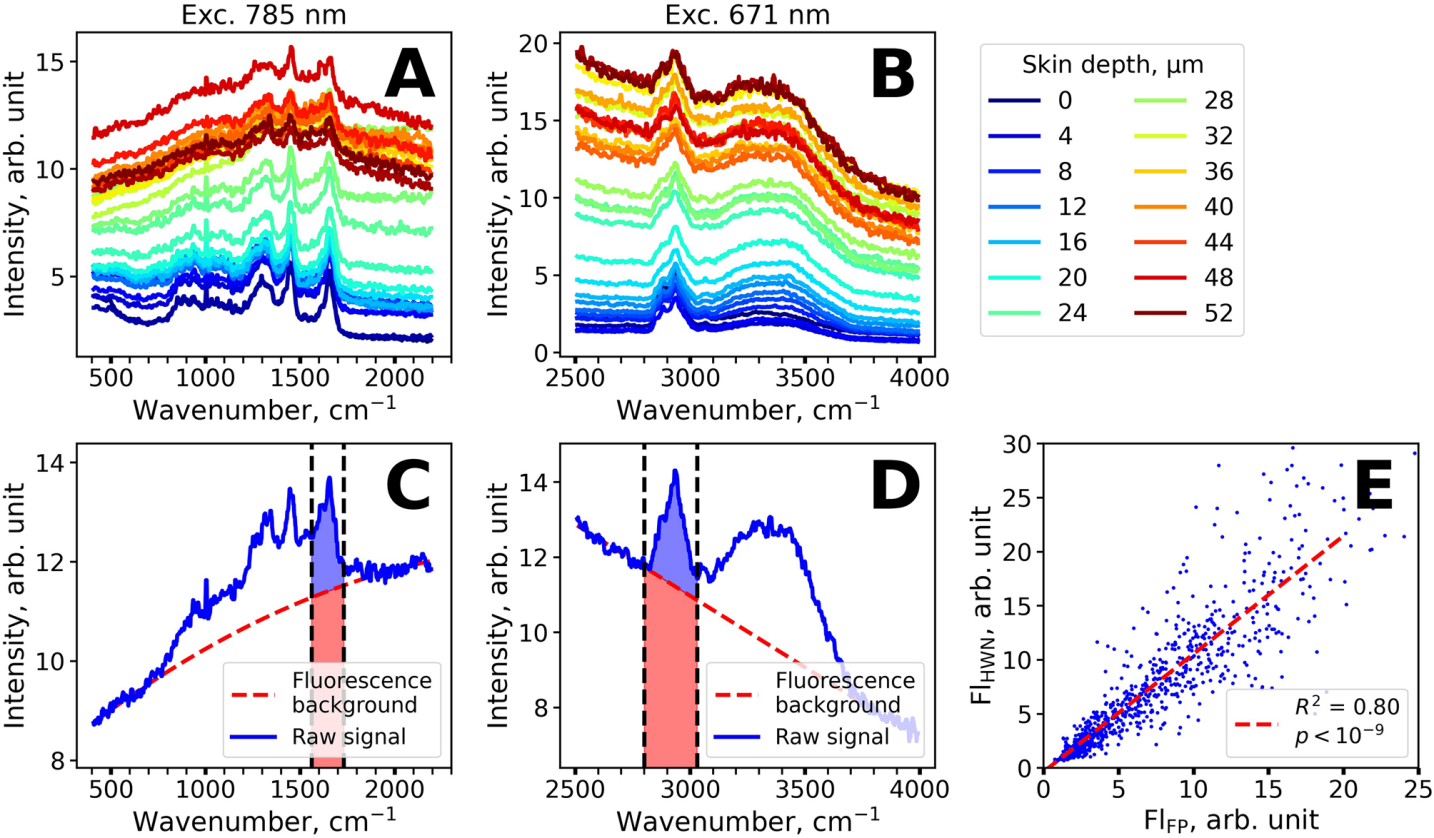
Characteristic depth-resolved Raman spectra of skin in vivo and examples of fluorescence background subtraction. (**A**,**B**) Depth-resolved Raman spectra of the human skin in the 400–2,200 cm^−1^ (**A**) and 2,500–4,000 cm^−1^ (**B**) ranges. Spectra are color-coded according to the measurement depth, ranging from 0 to 60 µm with 2 µm increments. (**C**,**D**). The examples of fluorescence background subtraction (red dashed line) for representative Raman spectra (blue line) in the 400–2,200 cm^−1^ (**C**) and 2,500–4,000 cm^−1^ (**D**) ranges. Red and blue areas filled in (**C**,**D**) represent fluorescence and Raman signal intensities used to estimate normalized fluorescence intensity (Fl-FP and Fl-HWN). (**E**) Correlation between the normalized fluorescence Fl-FP and Fl-HWN excited at 785 and 671 nm, correspondingly.

Figure 1A,B demonstrate the Raman-normalized spectra measured in vivo in the human skin of one volunteer (skin type II) at the depths varying from 0 to 60 µm with 2 µm increments. As it can be seen, the Raman signal is superimposed with a fluorescence background at all depths both in the FP (Fig. 1A) and HWN (Fig. 1B) regions that is typical for skin and biological tissues^23^.

Figure 1C,D demonstrate typical examples of the fluorescence background subtraction in the FP (Fig. 1C) and HWN (Fig. 1D) regions. The fluorescence signal averaged over the 1,550–1,720 cm^−1^ (FP region, red area in Fig. 1C) and 2,800–3,000 cm^−1^ (HWN region, red area in Fig. 1D) were normalized by the averaged Raman signal in the same wavenumber regions (blue area at Fig. 1C,D). The described averaged fluorescence intensities in FP and HWN normalized by Raman signal were further used for subsequent analysis, and hereinafter will be referred as Fl-FP and Fl-HWN.

It was observed that the calculated normalized fluorescence Fl-FP and Fl-HWN excited at different wavelengths (785 nm and 671 nm) were strongly correlated (Fig. 1E, linear approximation *R*^2^ = 0.78, *p* < 10^–9^), suggesting that the same fluorophores are responsible for fluorescence at both excitations. This suggestion is reasonable for melanin, which is characterized by the broadband absorption covering the red/NIR spectral range. From the depth-resolved fluorescence spectra shown in Fig. 1A,B, it can be seen that the NIR fluorescence signal substantially increases at ≈ 40 μm depths. This finding also suggests that the major source of the observed fluorescence is melanin, which is typically localized in the basal layer (at the depths of about 40 µm for the volar forearm skin^7^). As the fluorescence values Fl-FP and Fl-HWN were strongly correlated, we have further focused on the examination of the FP region.

### Assessment of melanin Raman bands in the skin: spectral decomposition

**Figure 2:**
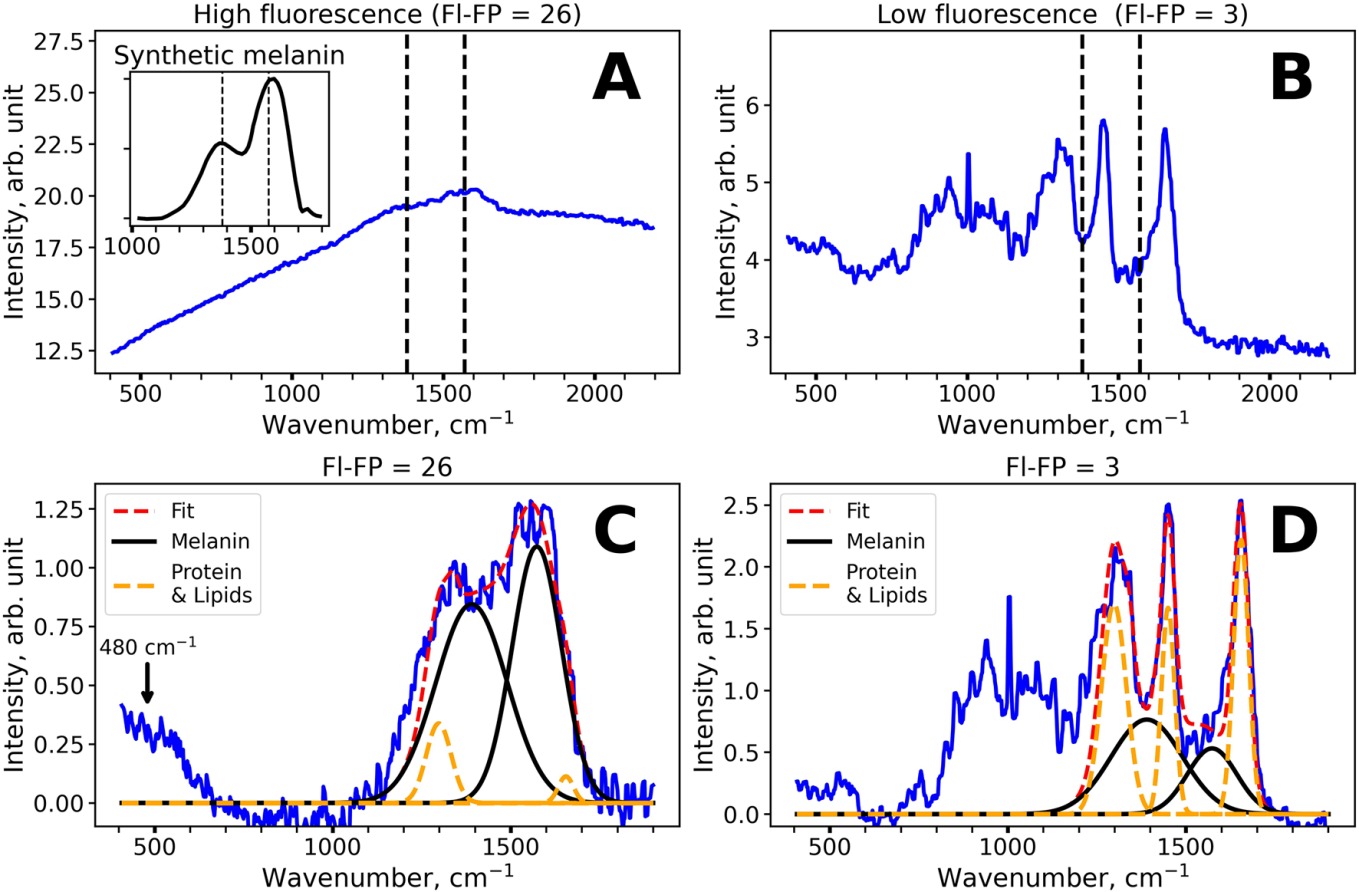
(**A**,**B**) Typical Raman spectra of the human skin in the FP region observed for high (Fl-FP = 26, (**A**) depth 36 µm) and low (Fl-FP = 3, (**B**) depth 18 µm) background fluorescence intensity. The inset in (**A**) demonstrates the Raman spectrum of synthetic melanin (M8631, Sigma), digitized from^41^. (**C**,**D**). The results of the decomposition of the Raman spectrum in the 1,200–1,800 cm^−1^ range for the samples with high (Fl-FP = 26, **C**) and low (Fl-FP = 3, **D**) fluorescence intensity. High intensities of lipid and protein bands at 1,298, 1,450 and 1655 cm^−1^ are observed for low fluorescence spectra (**D**), while the predominance of melanin bands at 1,380 and 1,570 cm^−1^ is observed for the spectra with high fluorescence background (**C**). Arrow in (**C**) denotes the melanin band located at ≈ 480 cm^−1^.

To assess the impact of melanin to the Raman signal formation, we performed decomposition of the Raman spectra (after fluorescence subtraction) in the 1,200–1,800 cm^−1^ region using Gaussian functions. As it can be seen in Fig. 2A,B, the spectral band shape alters significantly for the spectra with different fluorescence intensity (Fl-FP). The Raman spectra with high fluorescence background (Fl-FP > 15) (Fig. 2A,C) could be nicely described by the twin peaks of melanin with maxima near 1,380 and 1,570 cm^−1^. Indeed, these spectra are similar to that of melanin in vitro^41^ (see the inset in Fig. 2A). At the same time, the spectra with low fluorescence background exhibited three major bands at 1,298, 1,450 and 1655 cm^−1^, mainly corresponding to the vibrations of lipids and proteins of the skin^50^.

Hence, to determine the melanin content, we decomposed the Raman signal using intense Raman bands of lipids and proteins at 1,298, 1,450 and 1,655 cm^−1^ and two melanin-related bands at 1,380 and 1,570 cm^−1^. The approximation was performed using multiple Gaussian functions with fixed center positions and FWHMs, so only the amplitudes were varied. The FWHM values for the Raman bands of proteins and lipids at 1,298, 1,450 and 1655 cm^−1^ were fixed to 80, 45 and 50 cm^−1^ correspondingly. Since the melanin-related Raman bands are broadened due to its heterogeneous nature, FWHM values for the bands with centers at 1,380 and 1,570 cm^−1^ were fixed to 235 and 165 cm^−1^, in accordance with the previous reports^40–42,44^. The ratios between amplitudes of Gaussians were not fixed intentionally, as melanin bands are known to be sensitive to the chemical environment, excitation wavelength, etc. and were considered to be of interest for further analysis. The decomposition results of the Raman spectra with high (Fl-FP = 26) and low (Fl-FP = 3) fluorescence background are presented in Fig. 2C,D, respectively. The use of more peaks that would take into account other molecular components of the skin makes the approximation unstable. Moreover, it would not lead to a substantial increase in fitting accuracy because the proposed algorithm exhibited high approximation quality with R^2^ = 0.960 ± 0.018 within fitting range (1,200–1,800 cm^−1^), i.e. only 4% of the variance in the Raman spectra were unexplained by this fitting procedure.

We also verified the proposed fitting procedure by calculating pairwise correlation coefficients of the determined Gaussian amplitudes (Fig. SI1). The high correlation between the intensities of the Raman bands corresponding to lipids and proteins *I*_1298_, *I*_1450_, *I*_1655_ was observed (R^2 ^≈ 0.9), while the intensities of these bands did not correlate with the amplitudes of the melanin bands *I*_1380_ and *I*_1570_.

Besides the characteristic twin peaks located near 1,380 and 1,570 cm^−1^, the less intense broad band at about 480 cm^−1^ can be observed in the Raman spectrum of melanin^43,44^. In the skin spectra, where amplitudes of melanin-related bands substantially exceeded the sum of amplitudes of proteins and lipids Raman bands, we also observed the band located at ≈ 480 cm^−1^ region (Fig. 2C). The presence of this band can serve as an additional argument that melanin is the source of the observed fluorescence and form the skin Raman signal.

### Depth profiles of fluorescence and Raman signal

**Figure 3:**
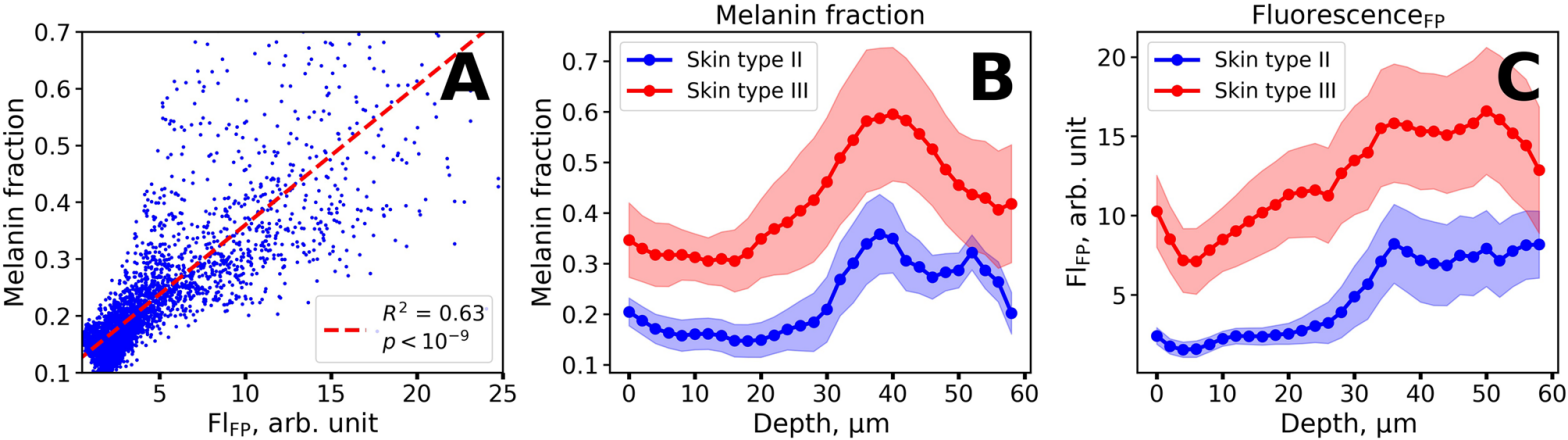
(**A**) Scatter plot of the melanin fraction estimated using Raman spectral decomposition in the 1,200–1,800 cm^−1^ range and NIR-excited fluorescence (Fl-FP). (**B**) Typical depth profiles of the melanin fraction estimated using Raman spectral decomposition for two volunteers with skin type II (blue) and III after sun exposure (red). (**C**) Typical depth profiles of fluorescence intensity (Fl-FP) values for volunteers with skin type II (blue) and III after sun exposure (red). Central lines represent mean values and color corridors represent standard deviation estimated using all depths profiles acquired for a volunteer.

We have investigated the interconnection between the melanin concentration, as obtained from the Raman spectral decomposition, and fluorescence background intensity. The fraction of melanin was calculated as the ratio of the sum of the amplitudes of the melanin-related bands to the sum of the amplitudes of all lines in the decomposition in the 1,200–1,800 cm^−1^ range (Fig. 2C,D). Figure 3A demonstrates a statistically significant correlation between the calculated melanin fraction and Fl-FP values (R^2^ = 0.63, *p* < 10^–6^). However, considerable variation in the melanin fraction in spectra with high fluorescence (Fl-FP > 10) can also be noticed. We addressed this issue by analyzing the depth dependences of both fluorescence and melanin Raman signals.

In Fig. 3B,C the representative depth profiles of the melanin fraction and Fl-FP are shown, calculated for one volunteer with skin type II and one volunteer with skin type III after sun exposure averaged over 6 depth-resolved profiles each. It can be noted that a lower melanin fraction corresponds to lower values of the fluorescence intensity (blue curve in Fig. 3B,C). In some cases, high Fl-FP (Fl-FP ≈ 10) and melanin fraction (≈ 0.4) can be detected at small depths (≈ 10 µm), corresponding to the *stratum corneum*. This was observed for the volunteers with skin type III after sun exposure. At a depth of about 35–40 µm both melanin fraction and Fl-FP values demonstrated a gradual increase, while at larger depths, the melanin fraction decreased. Depth profiles of melanin fraction and Fl-FP averaged within subgroups of volunteers with skin type II and III are presented in Fig. SI2. It was found that averaging over multiple volunteers could oversmooth observed distributions due to differences in epidermis thickness of volunteers.

The observed depth dependencies of the melanin fraction and Fl-FP values at depths exceeding 35 µm can be explained as follows. At the depths of ≈ 35–40 µm the basal layer is located where melanocytes are usually localized, hence, melanin in the basal layer is the source of the observed local maximum of the melanin fraction and stepwise increase in Fl-FP values at ≈ 35 µm. The decrease in the melanin fraction at larger depths (> 40 µm) can be explained by the crossing of the dermal–epidermal junction when focusing deeper into the skin. The stepwise behavior of fluorescence intensity suggests that at depths exceeding 40 µm the fluorophores located in the papillary dermis, e.g. collagen and elastin, can contribute to the overall fluorescence signal; therefore, the decrease of the fluorescence signal with depth is shallower than the decrease in the melanin fraction. In Figure SI3, we presented the Raman spectra for the depth profiles shown in Fig. 3B,C for the basal layer (35 µm, Fig. SI3A,C) and papillary dermis (55 µm, Fig. SI3B,D). One can see the predominance of the intensity of the melanin twin peaks in the 35 µm spectra, while proteins make a larger contribution to the spectra acquired at 55 µm.

### Assessment of melanin Raman bands in the skin: non-negative matrix factorization

**Figure 4:**
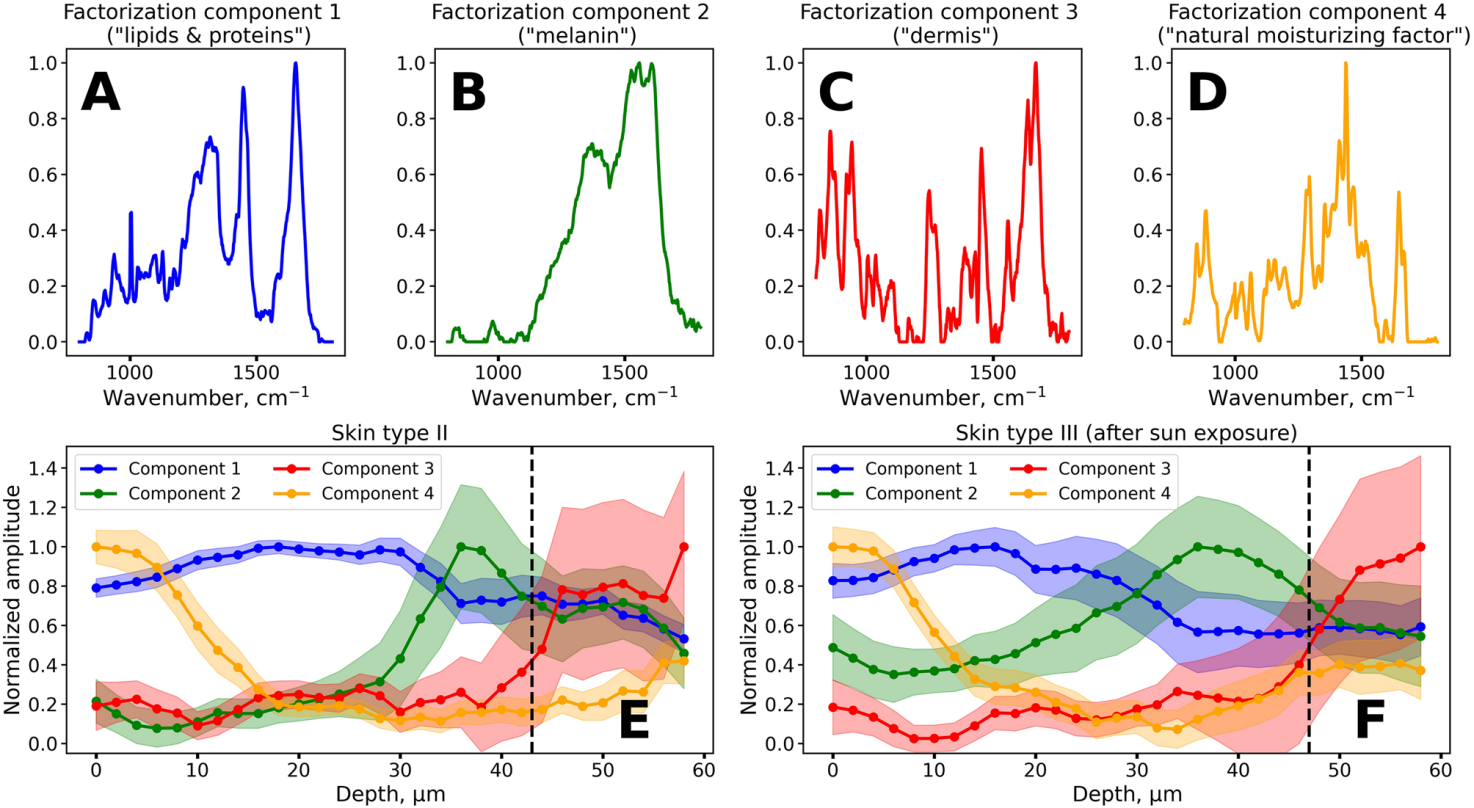
The results of the non-negative matrix factorization procedure applied to the Raman spectra of the skin. (**A**–**D**) Output spectral components, obtained from the factorization procedure. (**E**,**F**) Typical depth profiles of the amplitudes of decomposition components for the volunteers with skin type II (E) and skin type III after sunlight exposure (**F**). Vertical dashed line in (**E**,**F**) indicates the position of the dermal–epidermal junction. The spectral components and amplitude profiles are normalized by their maximum value for visibility.

To further confirm the results of melanin localization, we applied the non-negative matrix factorization procedure to the depth-resolved Raman spectra. Non-negative matrix factorization procedure is an unsupervised machine learning technique, which allows finding representation of the input matrix with non-negative elements as a product of two non-negative matrices of lower rank^51^. Vectors of the output matrices can be deduced as independent spectral components and their weights, with which they contribute to the initial Raman spectrum. This procedure is also suitable for the analysis of Raman spectra and the recovery of Raman spectra of individual constituents in a complex system^52^. In this work, the input matrix was composed of ≈ 2000 Raman spectra in the 800–1,800 cm^−1^ spectral range with the subtracted fluorescent background taken in equal proportions for all depths. Each Raman spectrum was preliminarily normalized to the 0–1 range to satisfy the condition of non-negativity and to exclude the dependency of the observed Raman signal on depth. We made use of six components for decomposition, the spectra of the first four components are shown in Fig. 4A–D, while all the components and their depth profiles are presented in Figs. SI4, SI5.

The first two spectral components of the non-negative matrix factorization were notably similar to the input Raman spectra. The first component (Fig. 4A) exhibited three intense bands at 1,298, 1,450 and 1655 cm^−1^, which are characteristic for lipids and proteins and are present in Raman spectra with low fluorescence intensity (see e.g. Figure 2D), while the second component (Fig. 4B) was identical to the Raman spectrum of melanin (Fig. 2C).

The amplitude of component 1 (blue line in Fig. 4E,F) remains almost unchanged with depth. Its spectrum fits the Raman spectra of lipids and proteins. The amplitude of component 2, which resembles Raman spectra of melanin, exhibited similar depth behavior as the melanin fraction calculated from the Raman spectra decomposition (Fig. 3B). We also found out that the spectral features of the third component (Fig. 4C) are similar to collagen (Fig. SI6), which could explain the depth dependence of the amplitude of the third component, which is almost zero for depths less than 40 µm, and exhibits an increase at larger depths (Fig. 4E,F). The observed stepwise increase of its amplitude is caused by the transition from the epidermis to the dermis, where collagen is one of the main structural components. The observed differences in the position of the dermal–epidermal junction (Fig. 4E,F) could be caused by the differences in the epidermis thickness of volunteers.

The spectral features of the matrix factorization component 4 (Fig. 4D) demonstrate a number of similarities with the Raman spectra of exemplary natural moisturizing factor (NMF) molecules (Fig. SI7)^53,54^. Moreover, the obtained depth profiles of component 4 (Fig. 4E,F) show the highest concentration in the superficial *stratum corneum* depths, that is specific for NMF molecules^55^. These facts confirm our assumption that the matrix factorization component 4 can be attributed to the NMF molecules. However, further investigations are required to prove this statement.

### Simple ratiometric approach for the assessment of melanin, dermal–epidermal junction and NMF distribution from the Raman spectral depth profiles

The melanin contribution to the Raman spectrum in the 1,200–1,800 cm^−1^ range can be also estimated without any use of decomposition and fitting procedures. As it can be seen in Fig. 2A,B the melanin-related band at 1,570 cm^−1^ only partially superimposes with the band at 1655 cm^−1^. Thus, the melanin fraction in the Raman spectrum can be estimated as the averaged intensity of Raman band in the vicinity of the 1,570 normalized to the 1655 cm^−1^ band intensity of proteins. Figure SI8 presents the estimation of the melanin fraction as the ratio of intensities averaged in the 1,500–1,590 and 1,590–1,710 cm^−1^ regions and demonstrates that it is positively correlated (R^2 ^≈ 0.96) with the value obtained from the spectrum decomposition procedure described above.

We also aimed at finding some ratio that would be correlated with the amplitude of the third component and the fourth component obtained with non-negative matrix factorization, which provides information about the dermal–epidermal junction and, presumably, NMF (Fig. 4). For this, we calculated the ratios of Raman spectra intensities for all pairs of wavenumbers in the 800–1,800 cm^−1^ range and then estimated the R^2^ values of linear correlations between the calculated ratios and the amplitude of the third component obtained from non-negative matrix factorization procedure. In Figure SI9A the heatmap of R^2^ correlation coefficients is shown. The highest linear correlation (R^2^ = 0.59) was observed for the *I*_1244_/*I*_1298_ ratio, where intensities at the indicated wavenumbers were averaged over a region ± 5 cm^−1^ near the center line (Fig. SI9B). It is plausible that molecular source of such changes in Raman spectra is collagen, as it is the main component of the dermal extracellular matrix and has an intense C–N Raman band at 1,244 cm^−1^^53,56,57^, while lipids, which are located in the epidermis, are a source of the Raman band at 1,298 cm^−1^^50^ Hence, the *I*_1244_/*I*_1298_ ratio can be a useful tool to locate the dermal–epidermal junction from the Raman spectral depth profiles. We also note that in component 3 (Fig. 4C) additional collagen specific Raman bands at 855, 938 and 1,670 cm^−1^ are visible, confirming our hypothesis about its connection with collagen. This is in agreement with findings described in^58^, where differences in the ranges 800–1,000 cm^−1^, 1,250–1,300 cm^−1^ and in the 1655 cm^−1^ amide bands were observed for the dermal–epidermal junction and attributed to the Raman spectrum of collagen in the dermis.

The procedure used to find optimal intensity ratio correlated with the amplitude of the third component (“dermis”) was also applied for the determination of intensity ratio that characterizes NMF in the *stratum corneum* and is correlated with the fourth component of non-negative matrix factorization (Fig. 4D). The ratios of the Raman spectra intensities calculated for all pairs of wavenumbers in the 800–1,800 cm^−1^ range for the Raman spectra acquired at depths lower than 20 µm were linearly correlated with the amplitude of component 4 of non-negative matrix factorization. The heatmap of R^2^ correlation coefficients is shown in Fig. SI10A. The highest correlation (R^2^ = 0.83) was observed for *I*_1412_/*I*_1620_ ratio (intensities at chosen wavenumbers were averaged over ± 5 cm^−1^ spectral region, Fig. SI10B). High R^2^ values were also observed for the ratios calculated at 886 cm^−1^ and 956 cm^−1^, 1,316 and 1,436 cm^−1^—the indicated wavenumbers correspond to local maxima and minima of several amino acids contained in NMF^53^. In Table 2, we summarized the characteristic features of ratiometric indicators presented in the work.

**Table 2:** Summary of simple ratiometric values used in the work, their molecular sources and depth behavior.

Ratiometric indicator	Molecular source	Depth behavior	Related values
Fl-FP (Raman-normalized fluorescence)	Melanin and protein oxidation products in the epidermis and dermal fluorophores	Stepwise increase near the basal layer	–
$\frac{{I}_{1244}}{{I}_{1298}}$	Collagen in the papillary dermis	Stepwise increase near the dermal–epidermal junction	Non-negative matrix factorization component 3 (Fig. 4C)
$\frac{{I}_{1412}}{{I}_{1620}}$	Natural Moisturizing Factor in the *stratum corneum*	Localization and monotonous decrease in the *stratum corneum*	Non-negative matrix factorization component 4 (Fig. 4D)
$\frac{{I}_{1540}}{{I}_{1655}}$	Melanin in the epidermis	Local maximum near the basal layer	Spectral decomposition amplitudes and (Fig. 2) and non-negative matrix factorization component 2 (Fig. 4B)

### Spectral features of NIR excited fluorescence and its relation to melanin molecular properties

**Figure 5:**
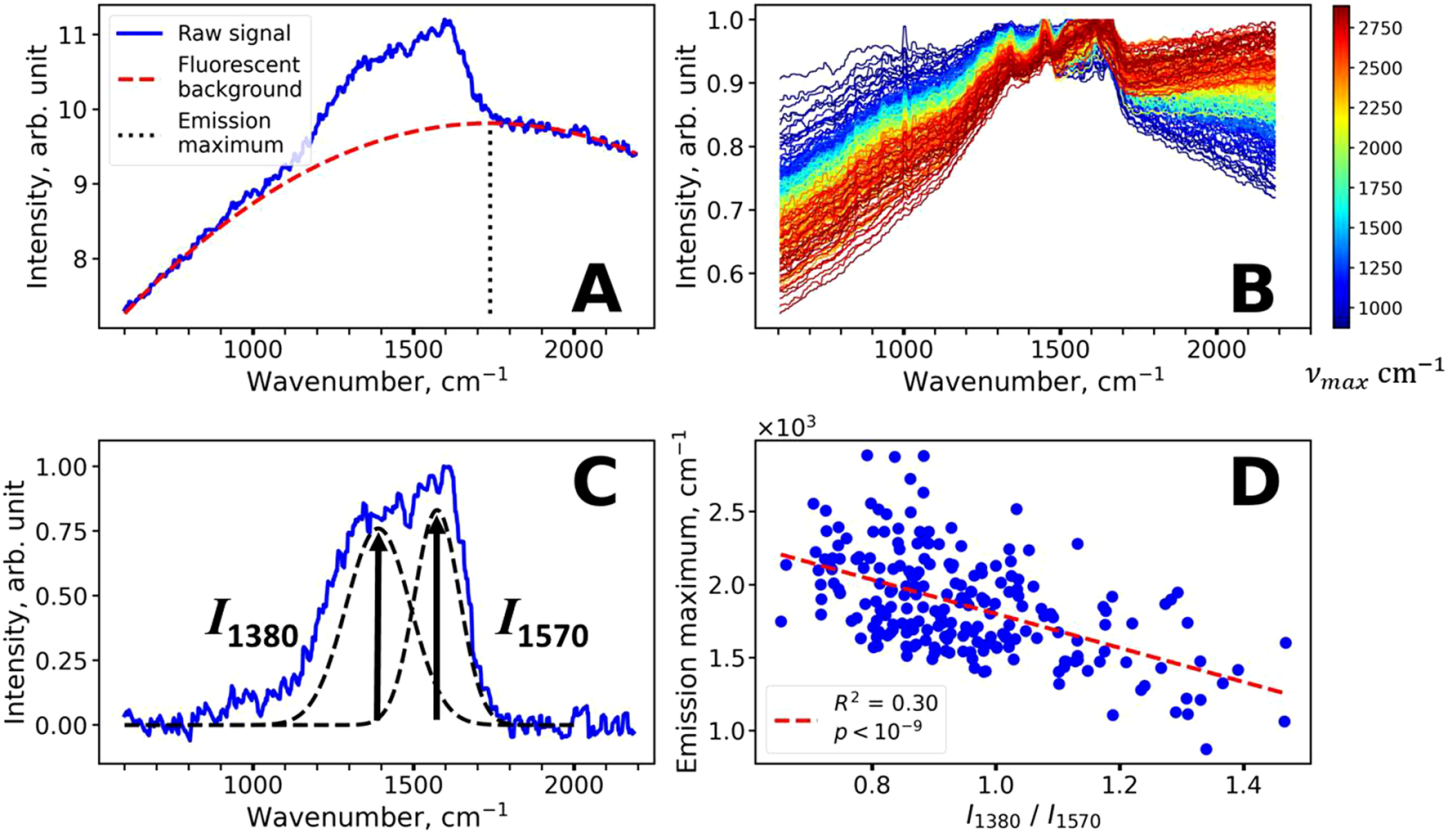
Assessment of spectral features of the NIR-excited fluorescence and its correlation with melanin-related Raman bands. (**A**) Illustration of the emission maximum estimation procedure. Fluorescence background was approximated by a 2^nd^ order polynomial, so the emission maximum could be calculated as a center of the parabola. (**B**) Representative spectra with high fluorescence (Fl-FP > 10) color-coded according to the position of the center of the parabola. (**C**) Typical Raman spectrum of the skin with high melanin content obtained for a volunteer with skin type III after sunlight exposure at a depth of 38 µm (*stratum basale*). Melanin twin peaks are denoted as dashed lines. (**D**) Correlation between the fluorescence emission maximum and the ratio of melanin Raman bands at 1,380 and 1,570 cm^−1^.

We assessed the position of the fluorescence maximum in the FP region by interpolation of the fluorescence background using the 2nd order polynomial function. This procedure allows determining the position of the emission maximum as the center of the parabola (Fig. 5A). We estimated the positions of the emission maxima for the spectra with high Fl-FP values (Fl-FP > 10,  ≈ 250 spectra in total, including depths from 10 to 60 µm). It was found that the position of the maximum varies significantly from 1,000 cm^−1^ (λ_max _≈ 850 nm) to 3,000 cm^−1^ (λ_max _≈ 1,030 nm). The representative spectra with Fl-FP > 10 used for calculation are presented in Fig. 5B and colored according to the position of the maximum of the fluorescence spectra.

Several hypotheses that could explain the spectral properties of the observed fluorescence signal were considered. Firstly, it could be assumed that the emission maximum may shift due to the self-absorption effect^59^. Melanin exhibits an exponential long-wavelength absorption extending to the NIR range. Hence, its increased absorption on the blue edge of the emission spectrum could lead to a more red-shifted fluorescence. Secondly, the variation of fluorescence spectral properties could be caused by the heterogeneity of melanin molecular properties.

The hypothesis about the role of self-absorption in the formation of fluorescence spectra band shape was verified as follows. The depth dependencies of the Raman intensities recorded in the range of 2,800–3,000 cm^−1^ excited at 671 nm and in the 800–850 cm^−1^ range excited at 785 nm were compared. These wavenumber regions selected for the 671 and 785 nm excitation correspond to the emission wavelengths of 833 nm and 839 nm respectively; thus, differences in attenuation of the Raman signals should be determined by the optical properties of the skin, i.e. differences in scattering and absorption, related to excitation, but not to emission. We did not observe significant differences between the fluorescence signal profiles acquired at these two excitation wavelengths (Fig. SI11). Hence, it could be argued that absorption does not cause significant changes in fluorescence band shape in vivo under the conditions used in this work.

In order to understand whether molecular properties and spectral properties of melanin fluorescence are related, we analyzed the relative intensity changes of the melanin Raman bands. For each Raman spectrum with Fl-FP > 10 the ratio of melanin band amplitudes *I*_1,380_/*I*_1,570_ was calculated (Fig. 5C,D) and plotted against the position of the maximum of the corresponding fluorescence spectrum. It was observed that the *I*_1,380_/*I*_1,570_ ratio was correlated with λ_max_ (R^2^ = 0.30, *p* < 10^–6^). We verified that the changes in the *I*_1,380_/*I*_1570_ ratio were not an artifact of fluorescence background subtraction. For this we varied the fluorescence background procedure by changing the left range over which the fluorescence background was subtracted from 500 ± 25 cm^−1^ to 950 ± 25 cm^−1^ with a 50 cm^−1^ step (Fig. SI12). Variations in *I*_1,380_/*I*_1570_ ratios and fluorescence emission maxima that were caused by such fluorescence background subtraction were less than the variations observed in the experiment. Thus, it can be assumed that the molecular properties of melanin manifested in the Raman spectra (evaluated as *I*_1,380_/*I*_1,570_ ratio) are also manifested in the spectral properties of its fluorescence (position of emission maximum). This difference in the amplitudes of melanin bands at 1,380 and 1,570 cm^−1^ might be presumably caused by different molecular properties of melanin such as oligomers packing, amount of oxidation and degradation, etc. and is of interest for future research.

It was also found that the molecular features of fluorophores contributing to fluorescence are manifested in the spectral properties of fluorescence. Namely, the fluorescence emission maxima determined from the fluorescence background shifts towards lower values with depth (Fig. SI13). We suppose that this could be explained by the finding that NIR fluorescence of collagen and elastin in the dermis (at depths > 40 µm) mainly could be blue-shifted in comparison to melanin fluorescence.

## Discussion

**Figure 6:**
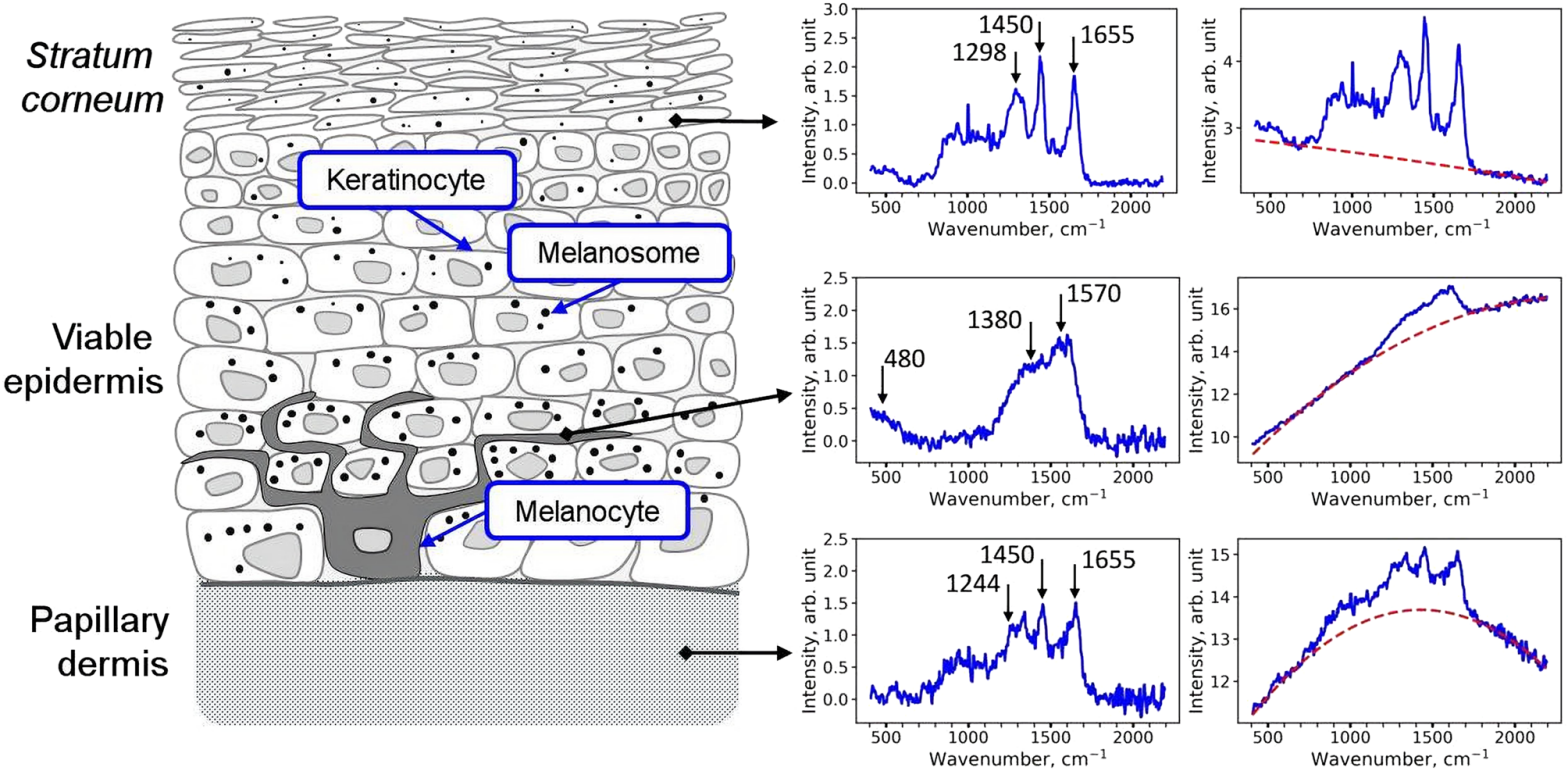
Schematic representation of the skin structure with an indication of typical Raman and fluorescence spectra corresponding to different layers.

Figure 6 presents representative Raman spectra for different layers of the skin. The *stratum corneum* layer has been extensively studied in the literature and distribution of lipids, water, NMF, DNA, keratin and carotenoids have been described in detail^46,47,50,53,60–63^. The maximum contribution from melanin was expectably found near the basal layer, where the characteristic twin peaks can be observed. However, depending on the skin type and exposure to sunlight, the admixture of melanin Raman bands to the Raman spectrum of *stratum corneum* can be detected and separated using the suggested approaches (Figs. 2, 3, 4). Spectral decomposition procedures also allowed separation between the impact of melanin and dermal constituents of the skin, which also displayed significant fluorescence upon NIR excitation that can be attributed to fluorescent cross-links and oxidation products in fibrillar proteins (collagens and elastin)^64,65^. Hence, the described procedures can be used to analyze Raman spectra depth profiles in the skin down to the papillary dermis.

The main idea of this work was to prove that melanin localization and depth distribution can be assessed from Raman spectra by disentangling the impacts of melanin and other skin constituents. The suggested approach thus allows estimation of the melanin fraction in different skin layers with molecular specificity that is its major difference from the battery of optical methods described in Table 1. While all of that methods are suitable for imaging of melanin in the basal layer, as well as for quantification of bulk melanin concentration, for the upper layers of the epidermis data interpretation is not that straightforward. That is, direct attributing of fluorescence in the upper epidermis to melanin is not possible, as other fluorophores such as keratin^66^ and proteins and/or lipids oxidation products^67^ may also contribute to the emission signal.

It should further be noted that the fluorescence intensity is prone to photobleaching, effectively reducing the fluorescence intensity over time. The origin of this effect is not completely identified. As the Raman bands are not subject to a photobleaching effect^49^, the normalization method using the ratio between fluorescence and Raman band intensities will be time-dependent and should be considered. However, due to the short exposure time used in this study, the authors propose that photobleaching plays a minor role.

When focusing on the basal layer, one can use a priori knowledge that melanin is the major fluorophore, and this insight is not applicable for the upper layers of the skin. In contrast to this, Raman microspectroscopy-based approach relies on the crosscheck of the signal origin by simultaneously analyzing the fluorescence and Raman spectra in each point. Hence, the first task where this approach can find application is the investigation of melanin fate and its redistribution in the epidermis upon stress conditions such as UV exposure^2,68^, oxidative and mechanical stress^11^, etc. Although this task is seemingly simple, the fact is that a number of questions about the melanin fate (such as the presence of “melanin dust” in the stratum corneum^13,14^), kinetics and mechanisms of its transfer in normal and stress conditions and the degradation pathways^7,11–14^ require additional studies, in which the suggested method could be useful.

Namely, after being transferred to keratinocytes through exo-/endocytosis of the melanosome core (melanocore)^69^, melanin’s concentration is regulated via the asymmetric distribution of melanin between daughter keratinocytes (the one remains in the basal layer and ‘inheriting’ most of the melanin, while the other destines to differentiate and stratify, and inherits a much smaller fraction of ‘maternal’ melanin)^11^ and, possibly, other mechanisms. In general, we can conclude that the degradation of melanin does not occur completely and it can be found in the upper layers of the epidermis, even in the superficial areas of the *stratum corneum*, and novel molecular-specific methods are required for studying the fate of melanin in the skin.

The second exciting possibility, which needs further investigation, is the characterization of the melanin structure in vivo using its Raman spectrum band shape. Surprisingly, despite its utmost significance, the optical properties of melanin are far from understood. This can be illustrated by the debates about the mechanisms of the absorption spectrum formation in melanin, where several hypotheses are considered^31,70,71^. The mechanisms of the melanin Raman spectrum formation are no less challenging, as the heterogeneous composition of the pigment, the arrangement of oligomers into aggregates and electronic interactions within the aggregates must be considered^42,70^. The striking similarity of the melanin Raman spectrum to that of disordered carbon systems such as graphene oxide^40,41,70^ makes this subject even more fascinating. Even on the superficial level of understanding, one can argue that the structure of melanin must be revealed in its Raman spectrum, e.g. in the ratio between the twin peaks^42^, which was presented in this work (Figs. 5C–D). On the other hand, the importance of assessing the melanin structure for melanoma characterization was well demonstrated in the works of Warren et al.^24,28–31^, where the oligomers stacking mode was shown to be manifested in the transient absorption properties. Based on the studies of synthetic melanins^42^, we believe that additional information about the melanin organization in vivo can be obtained from its Raman spectrum and, possibly, fluorescence spectrum, which exhibited surprisingly large variation of the band shape (the position of maximum varied from 860 to 1,000 nm upon 785 nm excitation) in our experiments (Fig. 5B). Together with the possibility to precisely localize melanin in the skin, this could open up new diagnostic ways of predicting and analyzing melanin-related disorders.

## Conclusion

In this work, the distribution of melanin in the epidermis was assessed using confocal Raman microspectroscopy by characteristic broad Raman “twin peaks” of melanin centered at ≈ 1,380 and ≈ 1,570 cm^−1^ as well as by NIR excited fluorescence of melanin. The suggested Raman-based approach allows estimating the melanin fraction in different layers of the skin with molecular specificity by disentangling the Raman spectrum of melanin in the 1,200–1,800 cm^−1^ range with the Raman signal of proteins, lipids and other constituents. It was found that multiple approaches, namely, fitting of Raman spectra by multiple Gaussian lines, unsupervised non-negative matrix factorization, as well as simple ratiometric indices can be successfully applied to gain insights into the melanin distribution in the epidermis, including the *stratum corneum*. The factorization procedure also provided information about the location of the dermal–epidermal junction and distribution of NMF.

The depth profiles of NIR excited fluorescence were found to correlate well with the melanin fraction as determined from the Raman spectra in the epidermis. However, high NIR fluorescence was also observed in the dermis, suggesting that it could be originated from other skin components such as oxidatively modified proteins.

We have also assessed the spectral band shape of the melanin-related NIR fluorescence. It was found that the position of the fluorescence emission maximum correlates with the ratio of the amplitudes of the melanin bands centered at ≈ 1,380 and ≈ 1,570 cm^−1^. Therefore, we believe that not only information about the distribution of melanin, but also insights into its molecular organization can be assessed by the combined Raman and NIR-fluorescence approach, which, in turn, can provide a new understanding of the behavior of melanin in healthy and pathological skin.

## Supplementary information

Supplementary information 1.

## Data Availability

The dataset analyzed during the current study is not publicly available due to ethical restrictions.
