# Supplementary material for: Melanin distribution from the dermal–epidermal junction to the stratum corneum: non-invasive in vivo assessment by fluorescence and Raman microspectroscopy
Source: Sci Rep. 2020 Sep 1;10:14374. doi: 10.1038/s41598-020-71220-6 (PMC7463016; doi:10.1038/s41598-020-71220-6)
Supplement: Supplementary file 1 — Supplementary information 1. [file 41598_2020_71220_MOESM1_ESM.docx]

## *Supplementary information*

## Melanin distribution from the dermal-epidermal junction to the *stratum corneum*: non-invasive *in vivo* assessment by fluorescence and Raman microspectroscopy

## B.P. Yakimov^1,2,3#^, E.A. Shirshin^1,3,5,#,*^, J. Schleusener^6^, A.S. Allenova^2,4^, V.V. Fadeev^1^, M.E. Darvin^6,*^

^1^M.V. Lomonosov Moscow State University, Faculty of physics, 1-2 Leninskie Gory, Moscow, 119991, Russia

^2^M.V. Lomonosov Moscow State University, Medical research and education center, Lomonosovsky Prospect 27/10, Moscow, 119991, Russia

^3^Institute for Regenerative Medicine, Sechenov First Moscow State Medical University, Trubetskaya 8-2, Moscow, 119991, Russia

^4^Sechenov First Moscow State Medical University, Division of Immune-mediated skin diseases, Trubetskaya 8-2, Moscow, 119991, Russia

^5^Institute of Spectroscopy of the Russian Academy of Sciences, Fizicheskaya Str., 5, 108840, Troitsk, Moscow, Russia

^6^Charité – Universitätsmedizin Berlin, corporate member of Freie Universität Berlin, Humboldt-Universität zu Berlin, and Berlin Institute of Health, Department of Dermatology, Venerology and Allergology, Center of Experimental and Applied Cutaneous Physiology, Charitéplatz 1, Berlin, 10117, Germany

# – both authors contributed equally to this work

^*^E.S.: [shirshin@lid.phys.msu.ru](mailto:shirshin@lid.phys.msu.ru), M. D.: [maxim.darvin@charite.de](mailto:%20maxim.darvin@charite.de)


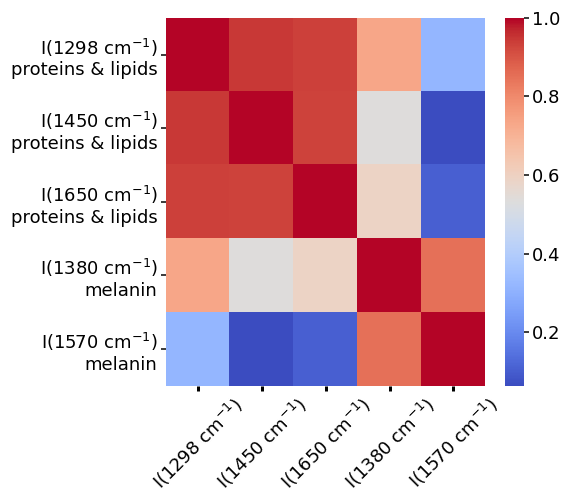


**Figure SI1.** Heatmap of the Pearson-*r* correlation coefficient between the Raman band intensities of decomposed Raman spectra. Intensities of lipids and protein related bands *I*_1298_*, I*_1450_*, I*_1650_ are highly correlated while poor correlation is observed between *I*_1298_*, I*_1450_*, I*_1650_ and intensities of melanin-related Raman bands *I*_1380_*, I*_1570_*.*


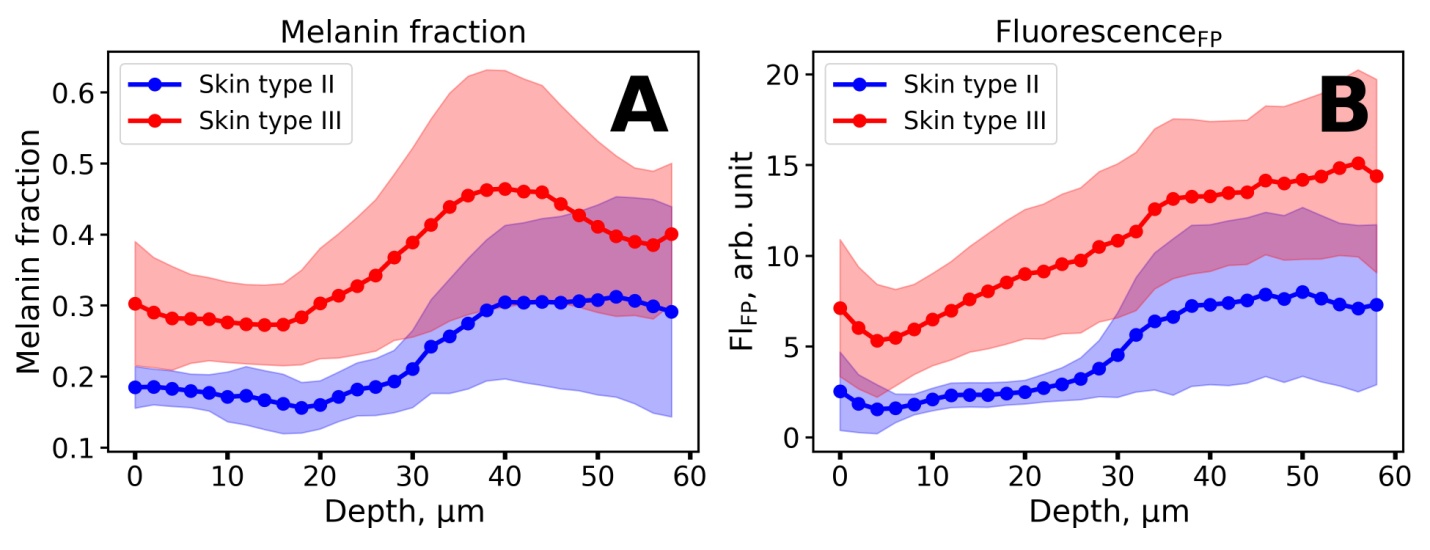


**Figure SI2**. A) Depth profiles of the melanin fraction estimated using decomposition of Raman spectrum B) Depth profiles of the fluorescence intensity (Fl-FP) averaged within subgroups of volunteers with skin types II (5 volunteers, blue) and III (5 volunteers, red). It can be seen that the distributions are smoothed due to the lack of accounting for the difference in the epidermal thickness.


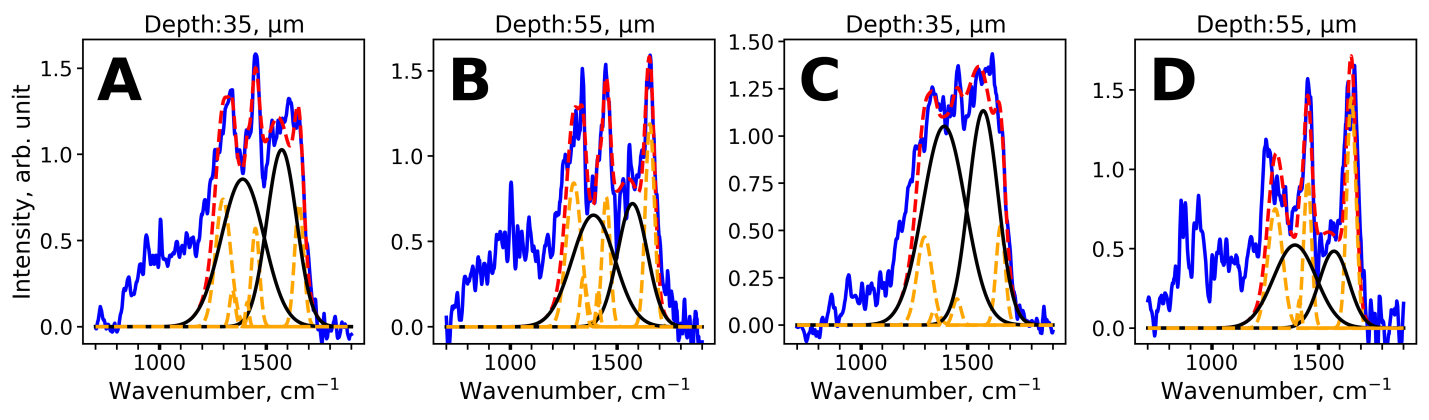


**Figure SI3.** A–B) Raman spectrum of skin at depths of 35 (A) and 55 µm (B) for profiles of a volunteer with skin type II. C–D) Raman spectrum of skin at depths of 35 (C) and 55 (D) µm for profiles of a volunteer with skin type III. A prevalence of melanin contribution is observed at 35 µm, where the basal layer is primarily located.


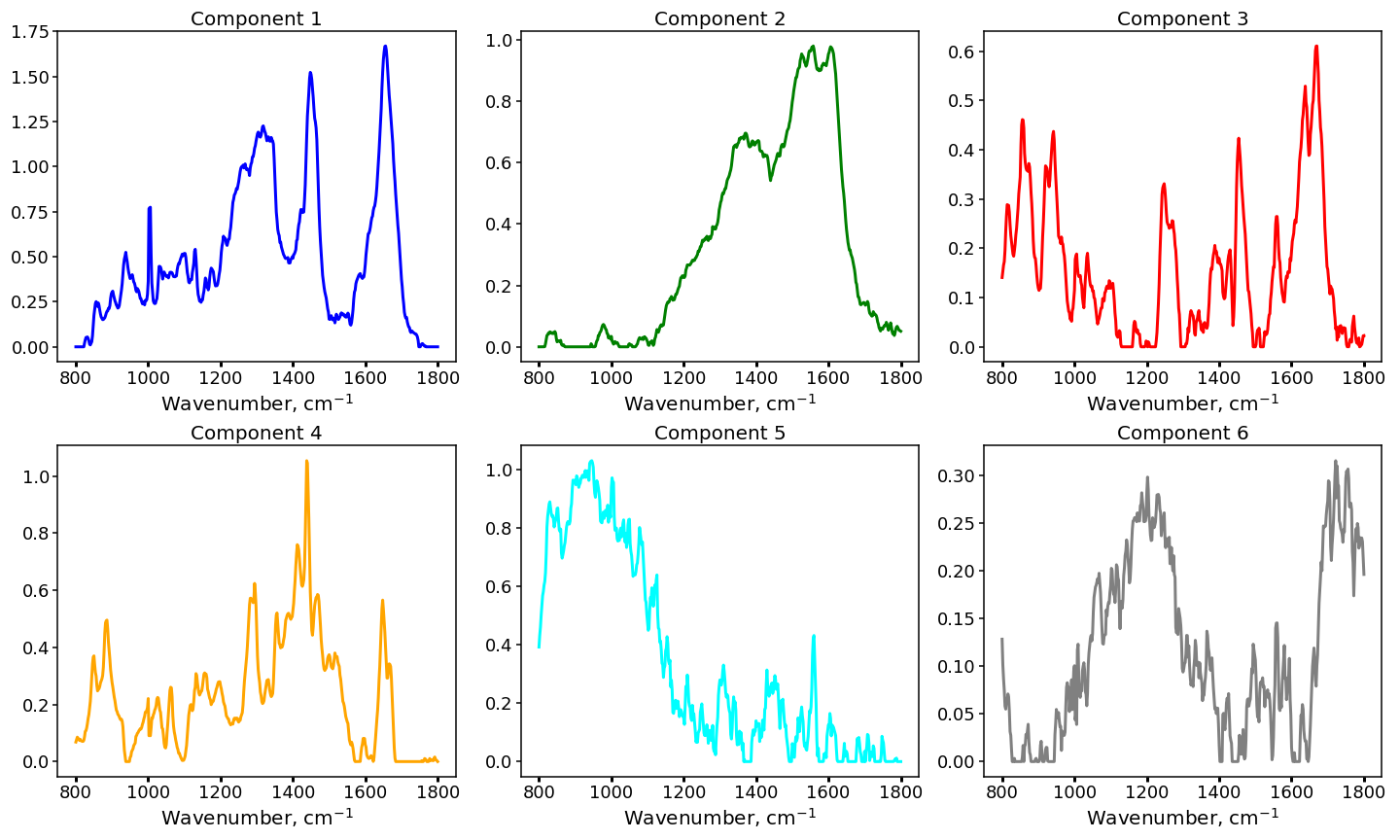


**Figure SI4**. Output components of the non-negative matrix factorization procedure.


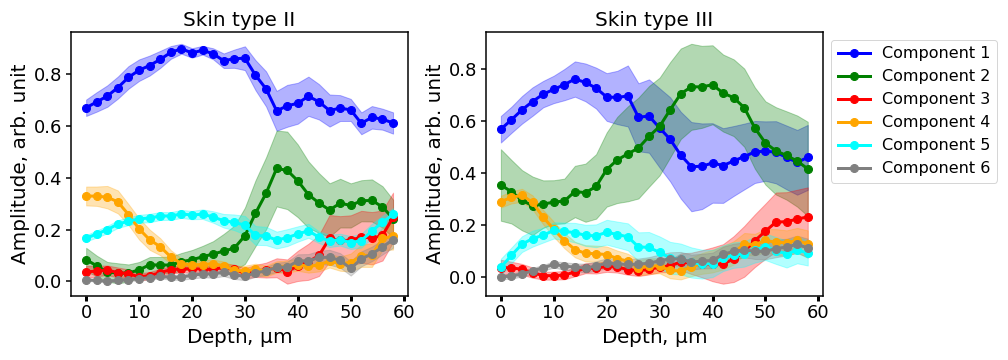


**Figure SI5.** Non-normalized depth-resolved profiles of amplitudes of the non-negative matrix factorization for skin type II and III after sun exposure.

**
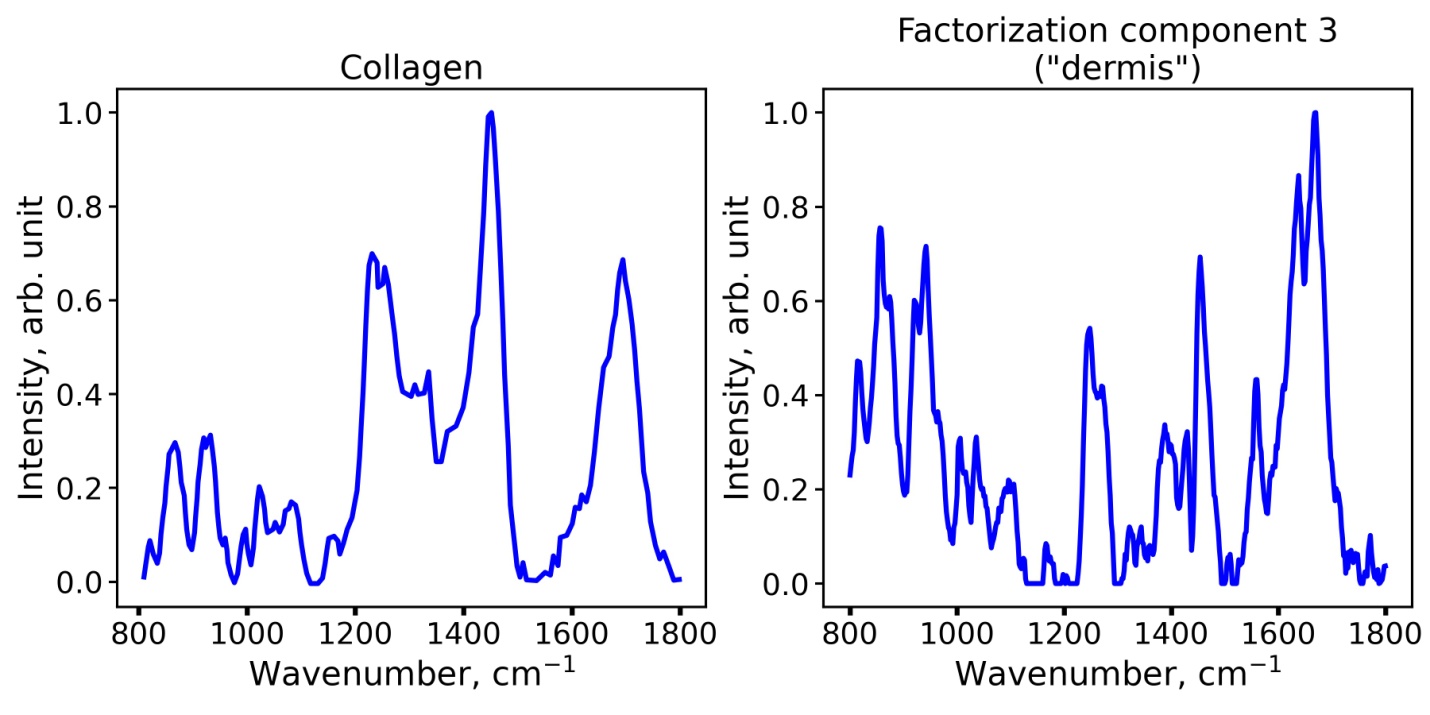
**

**Figure SI6.** (left) The Raman spectrum of collagen from [1] (right) The spectrum of component 3 of the non-negative matrix factorization procedure.


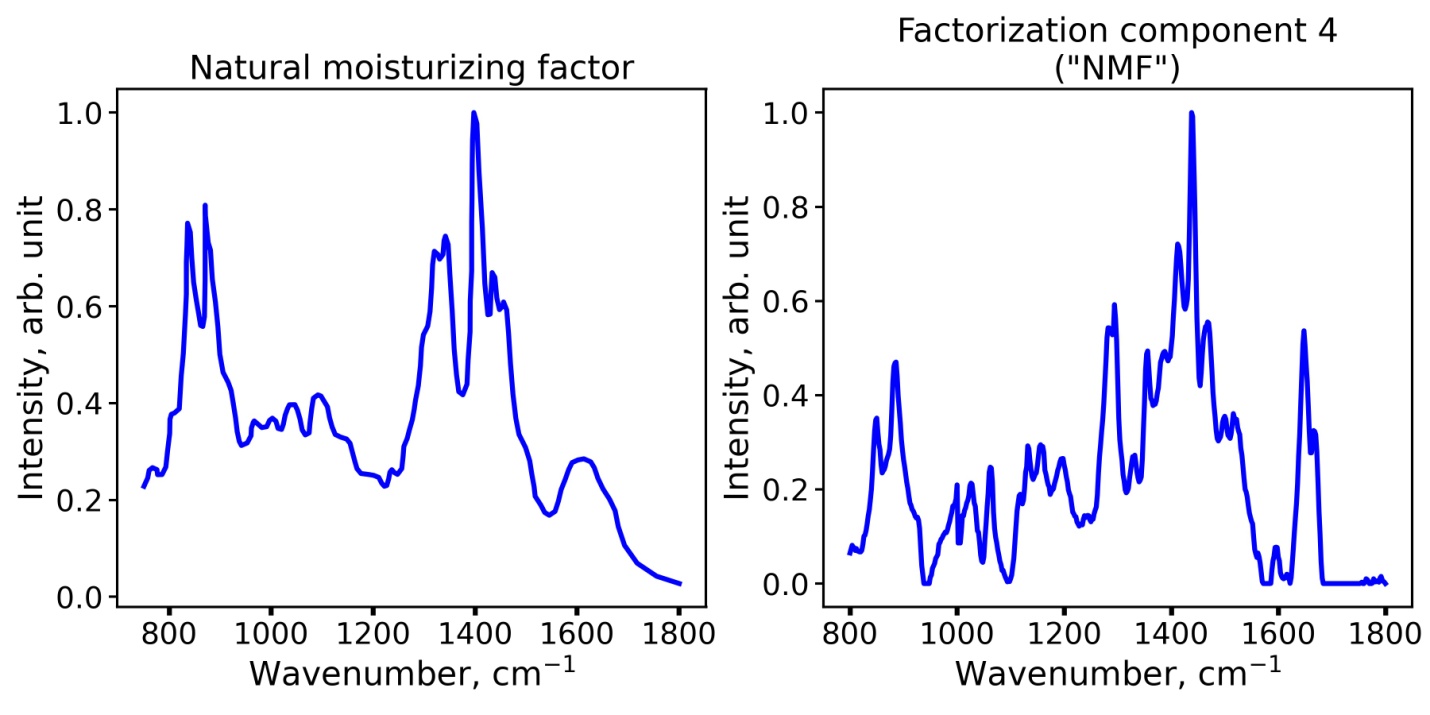


**Figure SI7.** (left) The Raman spectrum of NMF from [2] (right) The spectrum of component 4 of the non-negative matrix factorization procedure.


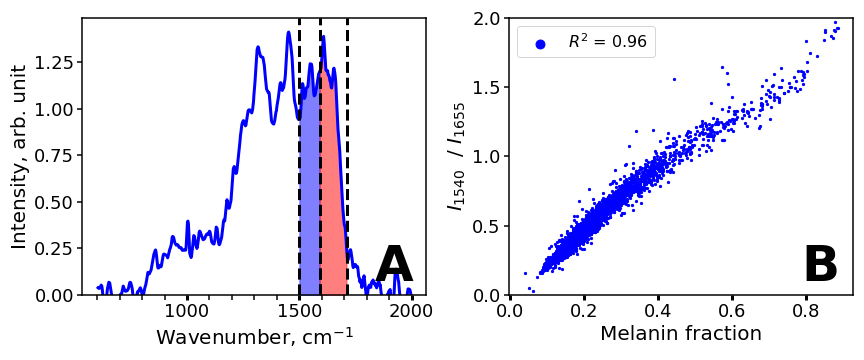
**Figure SI8.** A) Example of a Raman spectrum of skin (depth 28 µm) used for the estimation of the melanin contribution to the spectrum as ratio of intensity averaged over the 1500–1590 cm^-1^ range (*I*_1540_, blue area, melanin) to an intensity averaged over the 1590–1710 cm^-1^ range (*I*_1655_, red area, proteins). B) Correlation between the melanin fraction estimated using decomposition of Raman spectra using multiple peaks in the 1200–1800 cm^-1^ range and the ratio of averaged intensities *I*_1540_*/I*_1655._


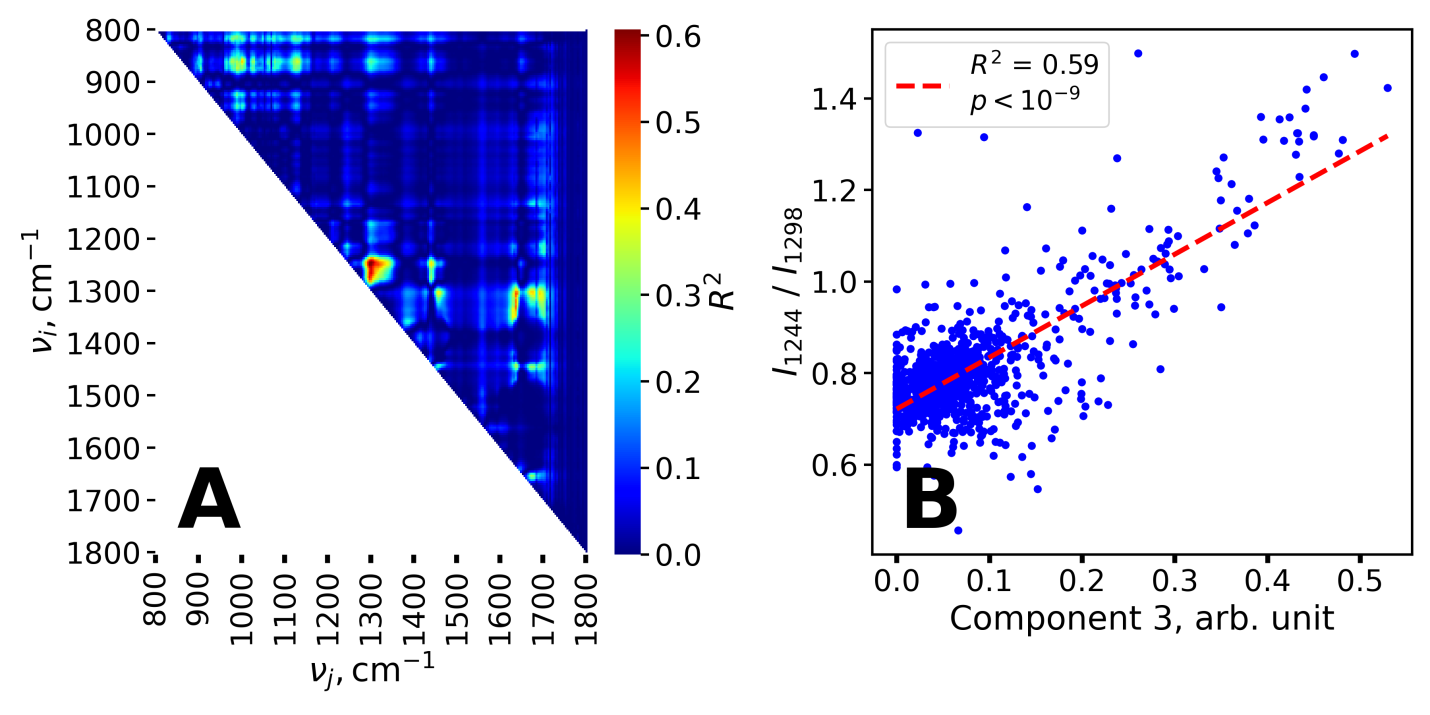


**Figure SI9.** A) Heatmap of R^2^ of linear correlation between intensities ratio $I(\nu_{i})/I(\nu_{j})$ of Raman signal and amplitude of the third (“dermis”) component of non-negative matrix factorization. B) Scatter plot of the correlation coefficient between amplitude of the third component of non-negative matrix factorization and the ratio *I*_1244_/*I*_1298_ with highest R^2^.


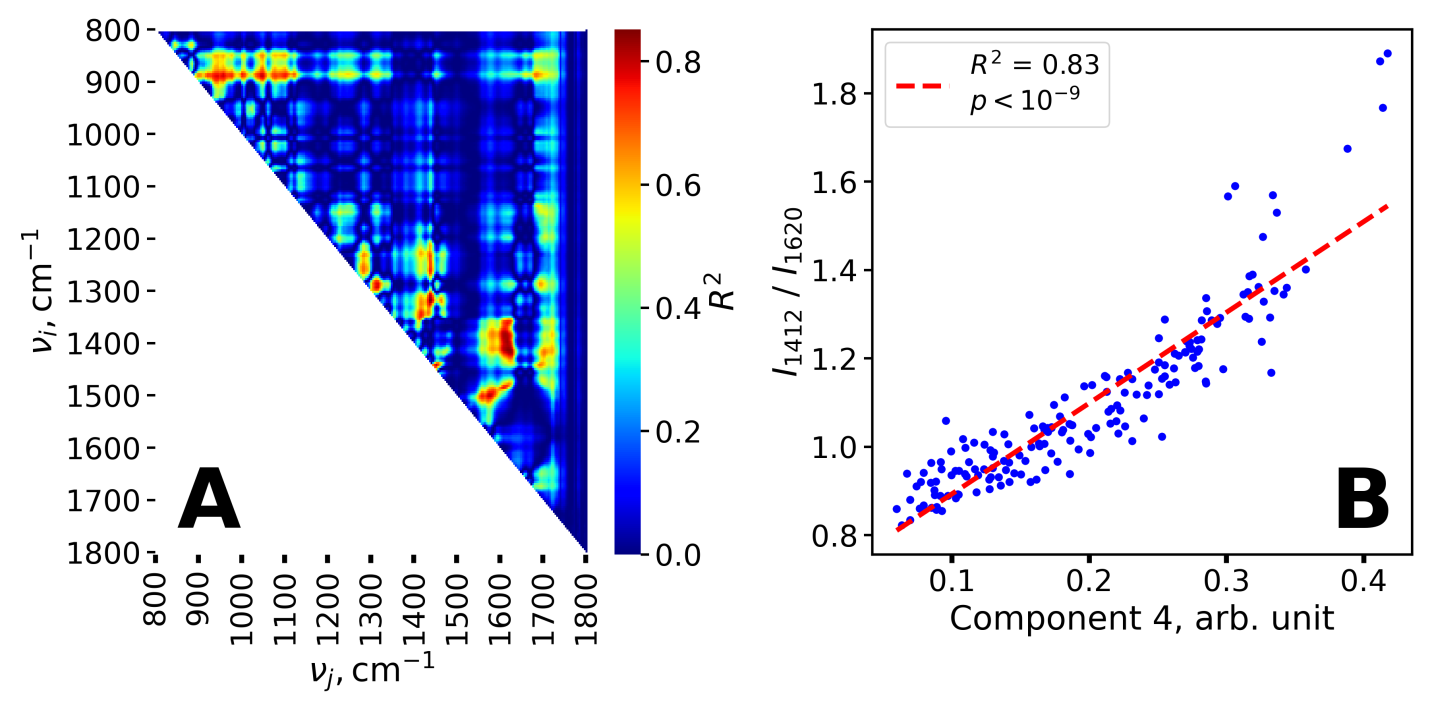


**Figure SI10.** A) Heatmap of R^2^ of linear correlation between intensities ratio $I(\nu_{i})/I(\nu_{j})$ of Raman signal and amplitude of the fourth (“NMF”) component of non-negative matrix factorization for spectra acquired at depth lower than 20 µm. B) Scatter plot of the correlation coefficient between amplitude of the third component of non-negative matrix factorization and the ratio *I*_1412_/*I*_1620_ with highest R^2^


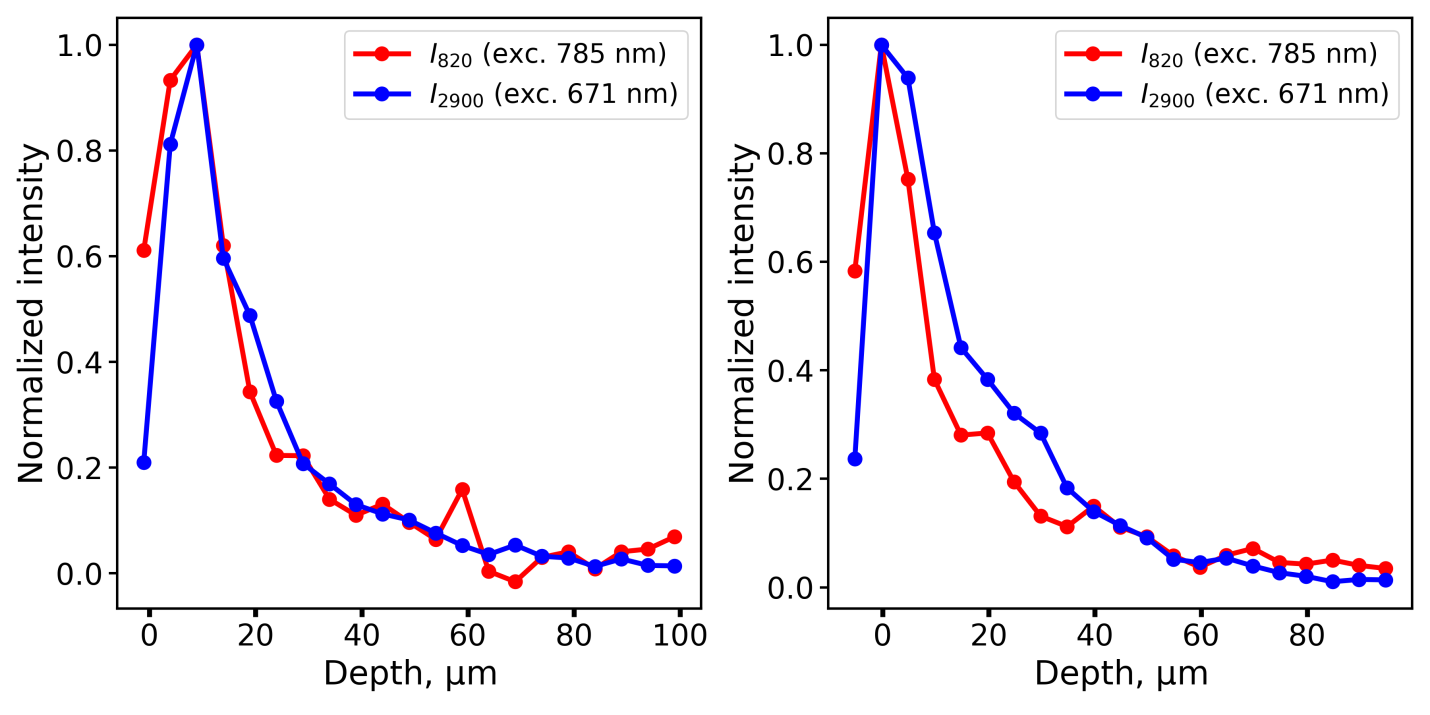


**Figure SI11.** Attenuation profiles of Raman signal at different excitation wavelengths for a volunteer with skin type III after sun exposure. Intensity values are normalized on maximum.

**
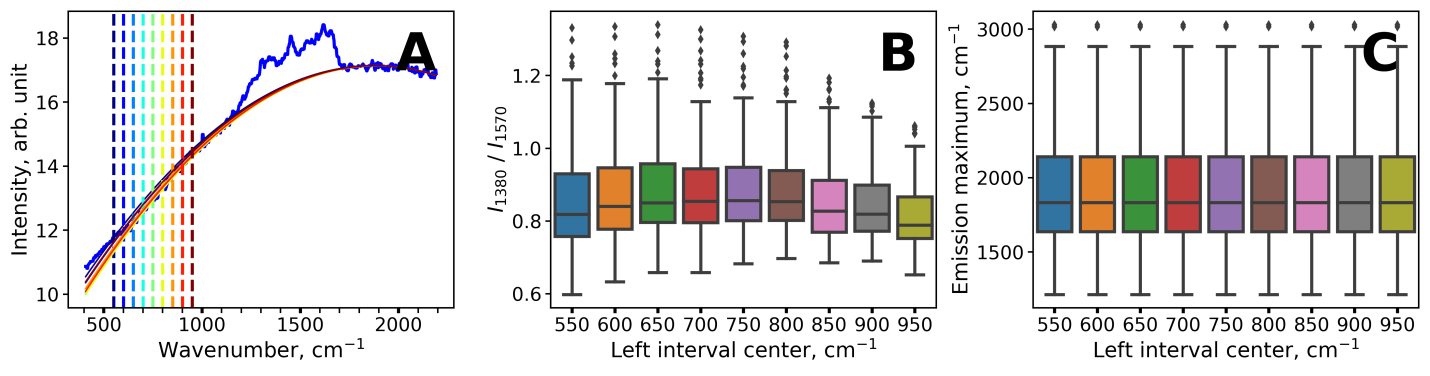
**

**Figure SI12.** Explanations regarding the variation of the background procedure. A) Typical Raman spectrum with high fluorescent background. Centers of the left interval, through which the interpolation of fluorescence background was carried out are denoted as colored dashed lines. B) Distribution of *I*_1380_/*I*_1570_ ratio for different positions of left interval through which the interpolation of fluorescence background was carried out. C) Position of fluorescence emission maxima estimated as the center of the 2^nd^ order polynomial used for the fitting of fluorescence background calculated for different positions of the left interval.


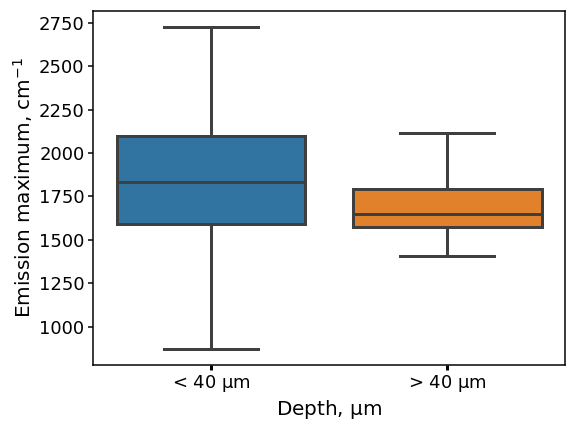


**Figure SI13.** Distribution of the emission maximum of NIR-excited fluorescence for spectra with intense fluorescence background (Fl-FP>10) acquired at depth lower and higher than 40 µm. The emission maximum for the group of spectra acquired at depths >40 µm demonstrates a statistically significant blue-shifted fluorescence (Kruskal-Wallis test *p*=0.01).

## References

1. Bergholt, M., Serio, A. & Albro, M. Raman Spectroscopy: Guiding Light for the Extracellular Matrix. *Front. Bioeng. Biotech.* **7**, 303, <https://doi.org/10.3389/fbioe.2019.00303> (2019).
2. Baclig, A. C. *et al.* Possibilities for human skin characterization based on strongly reduced Raman spectroscopic information. *J. Raman Spectrosc.*  **44**, 340–345, <https://doi.org/10.1002/jrs.4198> (2013).
